# The relationship between perceived intensive parenting and college students' academic achievement: the mediating roles of academic self-management and goal orientation

**DOI:** 10.3389/fpsyg.2026.1894707

**Published:** 2026-08-19

**Authors:** Yuan Zhou, Hsuan-Po Wang

**Affiliations:** Chinese International College, Dhurakij Pundit University, Bangkok, Thailand

**Keywords:** academic achievement, academic self-management, college students, goal orientation, perceived intensive parenting

## Abstract

In recent years, increasing parental involvement in children's education has attracted growing attention, particularly regarding how intensive parenting practices influence students' academic development and psychological characteristics. This study is grounded in social ecological systems theory and aims to explore the relationship between college students' perceived intensive parenting and their academic achievement, as well as to further examine whether perceived intensive parenting is associated with academic achievement through academic self-management and goal orientation. The study collected data through a questionnaire survey and conducted empirical analysis using a sample of 569 Chinese university students. The research results indicate that college students' perception of intensive parenting by their parents is associated with academic achievement, and this association is indirectly mediated by students' academic self-management abilities and goal orientation levels. This study provides new empirical evidence for understanding the relationship between parenting styles and the academic development of college students. It also offers valuable references for family education practices and university student management, helping educators, families, and related research fields to gain a more comprehensive understanding of the growth and development process of college students.

## 1 Introduction

For individual students, academic achievement is the fundamental basis for career development, social mobility, and the realization of personal value (Stadler et al., 2021). From a societal perspective, it is also the most tangible return on national human capital accumulation and educational investment (Li et al., 2021). However, student dropout and academic failure have become serious issues faced by universities worldwide (Behr et al., 2020; Long and Noor, 2023; ThinkImpact, 2023). Among them, Chinese students place particular emphasis on academic achievement, almost considering it the sole criterion for academic success (Yang and Soon, 2024). Academic achievement in this study primarily reflects university students' subjective perceptions of their academic accomplishments, encompassing multiple dimensions, including academic engagement, learning performance, interpersonal promotion, and objective achievement (Luo et al., 2023).

In recent years, college students' academic achievement has become a central focus in the higher education literature, with existing studies examining the factors associated with academic performance from multiple perspectives, including individual, psychological, school, and family factors (Alyahyan and Düştegör, 2020; Wang and Chen, 2025). College students are in a critical stage of being “legally adults but not yet independent” (LaFreniere, 2024). In this context, the academic development of college students not only requires individual effort but also relies heavily on the family as the fundamental environment for their growth (Zhou Q. et al., 2025). Therefore, examining the family factors that are related to college students' academic achievements is of extremely significant practical importance for the high-quality development of education and the overall development of students.

In the context of traditional Chinese family culture, where parents have high expectations for their children to achieve outstanding accomplishments, coupled with the increasingly intense educational competition of contemporary times, an intensive parenting style has emerged (Gu, 2021). This parenting style refers to parents investing a significant amount of time, energy, and resources in their children's growth, actively participating in their academic and daily lives (Faircloth, 2023). Intensive parenting is distinct from general parental involvement or excessive interference in learning (Yerkes et al., 2021). Prior research suggests that parental involvement or interference tends to emphasize control and supervision over the learning process, which may even restrict students' autonomy (Hartini et al., 2022). In contrast, intensive parenting places greater emphasis on substantial parental investment in time, emotional support, and resources (Lamprianidou et al., 2025). Existing studies have demonstrated that intensive parenting can facilitate students' academic progress (Liu, 2024; Poudel et al., 2024). For college students, continuous parental attention and active support can serve as a reliable source of backing (Kang, 2024). However, excessive parental interference may produce markedly different effects on college students' academic achievement (Xie et al., 2022). In this context, how perceived intensive parenting is associated with college students' academic achievement remains an issue that this study seeks to clarify.

In view of the growing importance attached to academic achievement, scholars have begun to examine its influencing factors from multiple perspectives. First, as an intrinsic driver of students' learning behavior, goal orientation plays a crucial role in academic achievement (Tang et al., 2021). Parenting styles, particularly the level of parental involvement and expectations, have a profound impact on the development of students' goal orientation, shaping whether they adopt a “goal acquisition orientation” or a “goal attainment orientation” in their learning (Lubis et al., 2022). Lu et al. (2022) tracked the first-year situation of 316 Chinese university students, and the results showed that students with a higher learning goal orientation had better academic achievement. By promoting deeper student engagement in learning, this orientation significantly improved academic achievement (Zare Pak Ziabari and Dabiri, 2023). Additionally, Hayek et al. (2022) indicate that individuals' intrinsic psychological traits play a crucial mediating role between parenting styles and academic achievement. Academic self-management refers to an individual's ability to plan, monitor, and adjust the learning process to achieve learning goals. It serves as an important bridge connecting the external environment with the individual's academic achievement (Stan, 2021). Research shows that a supportive environment can actively cultivate students' self-management skills (Martínez-López et al., 2024). Students with stronger self-management skills often achieve higher academic achievement (Lubis et al., 2022). Given these associations, this study aims to examine the relationship between college students' perceived intensive parenting and their academic achievement, and further investigate whether perceived intensive parenting is associated with academic achievement through academic self-management and goal orientation.

Jia et al. (2022) and Shi et al. (2021) suggested that only children tend to achieve better academic performance, which they attributed to their greater access to family educational resources. However, other studies have argued that interactions with siblings help develop cooperation and problem-solving skills, placing non-only children at no clear disadvantage, while the academic differences between the two groups gradually diminish after entering college (Li et al., 2021; Chan and Dai, 2023). Parental educational attainment is generally positively associated with college students' academic achievement, and this association may reflect that highly educated parents are better able to provide learning resources and educational support that facilitate their children's academic development (Wang and Panicker, 2024; Zaman et al., 2024). In addition, a higher frequency of parent–child communication is generally associated with better academic performance, although the quality of communication appears to be more important than its frequency, and excessively frequent or low-quality communication does not necessarily improve academic achievement (Schwanz et al., 2014; Weintraub and Sax, 2018; Fingerman et al., 2016; Zhang, 2020). Based on these observations, this study included only-child status, parental educational attainment, and weekly frequency of parent–child communication as control variables.

In the Chinese cultural context, close intergenerational ties and strong parental involvement often extend into emerging adulthood (Jiang and Zhang, 2025). Even after entering college, parents frequently remain involved in students' academic and career planning, making intensive parenting a relevant factor during this developmental stage (Wei et al., 2022). Based on the above reasoning, Perceived Intensive Parenting has a predictive effect on college students' academic achievement, and academic self-management and goal orientation play a mediating role in this relationship. The intensive parenting style is not only associated with academic achievement but also is indirectly associated with the final academic achievement through these intrinsic traits (Ye et al., 2024). Although existing research has preliminarily explored the pathway through which parenting styles are indirectly associated with academic achievement via goal orientation, further studies are still needed to verify the mechanisms underlying the specific relationship between intensive parenting styles and academic achievement at this stage.

## 2 Literature review and hypotheses

### 2.1 Ecological systems theory

Bronfenbrenner (1979) proposed Ecological Systems Theory, which posits that individual behavior is embedded within a series of interacting environmental systems. These systems comprise four nested levels, from the innermost to the outermost: the microsystem, mesosystem, exosystem, and macrosystem. Lubis et al. (2024) argued that these systems are interconnected and dynamically evolve over time in response to social and cultural changes, jointly shaping individuals' developmental trajectories. Ecological Systems Theory emphasizes the interaction and adaptation between individuals and their environments. The microsystem consists of settings in which individuals directly participate, such as the family and school; the mesosystem encompasses the interrelationships among communities and institutions in which individuals are involved; the exosystem includes broader contextual factors, such as government policies and sociocultural conditions, which are indirectly associated with individuals despite their lack of direct involvement; and the macrosystem represents the overarching societal value and belief systems (Kaushik et al., 2023). This theory provides a comprehensive explanation of how multiple environmental systems interact and coordinate to be associated with individual development, thereby deepening the understanding of the relationship between individuals and their environments while highlighting the integrated influence of multilevel environmental factors on human development (Bronfenbrenner, 1979).

Ecological Systems Theory serves as the core theoretical foundation of the present study because its multilevel and dynamic analytical perspective closely aligns with the conceptual framework of this research in three key aspects. First, the hierarchical structure of the theory corresponds closely to the systematic relationships among the study variables. The core variables examined in this study, namely perceived intensive parenting, academic self-management, goal orientation, and academic achievement, are not isolated individual characteristics but are embedded within multiple environmental contexts, including the family, school, and broader sociocultural environment (Yang and Oh, 2024). The four-level framework of Ecological Systems Theory not only accommodates the sequential pathway from family parenting practices (the central component of the microsystem), through individual psychological mechanisms (academic self-management and goal orientation), to academic outcomes (academic achievement), but also provides a comprehensive theoretical framework that incorporates the potential influences of the mesosystem and the macrosystem on this developmental process (Mulisa, 2019). Consequently, the theory offers a robust foundation for examining the complex interactions among the study variables.

Second, the core assumptions of the theory are well suited to the developmental characteristics of college students. Emerging adulthood is a unique developmental stage characterized by the coexistence of dependence and independence. During this period, college students continue to have substantial connections with their family environment while simultaneously becoming increasingly integrated into broader ecological systems, including educational institutions, peer networks, and the wider sociocultural context. In this context, the interactions between individuals and their environments become increasingly complex and dynamic (García-Mendoza et al., 2024). The emphasis of Ecological Systems Theory on dynamic interactions among environmental systems and individual agency provides an appropriate explanation of how parenting practices shape students' internal psychological mechanisms, which are subsequently associated with their academic performance within higher education settings. This perspective effectively addresses the limitations of traditional parenting theories, which primarily focus on parent–child interactions within the family while overlooking the interconnected influences of multiple environmental systems (Wang et al., 2024).

Third, the internal logic of Ecological Systems Theory provides a strong theoretical basis for the mediating hypotheses proposed in this study. The central assumption of the theory is that the microsystem constitutes the most immediate source of influence on individual development (Wang et al., 2023), and that environmental factors are associated with developmental outcomes through individuals' internal cognitive and behavioral processes rather than exerting direct influences (Li et al., 2025). Specifically, the theory suggests that the family, as the core component of the microsystem, is associated with developmental outcomes indirectly by shaping individuals' internal psychological mechanisms, such as self-regulation and goal cognition, rather than through direct effects (Chinyoka and Naidu, 2014). This theoretical proposition closely corresponds to the mediation model proposed in the present study, in which perceived intensive parenting is associated with academic achievement through academic self-management and goal orientation.

Based on the hierarchical logic and core assumptions of Ecological Systems Theory, the theoretical model proposed in this study can be clearly explained. Intensive parenting, characterized by high levels of parental investment and control, represents the most immediate and influential environmental factor within the family microsystem experienced by college students and serves as the core independent variable in the model (Jeynes, 2023). According to Ecological Systems Theory, the quality of interactions between individuals and their microsystem determines the direction and level of their psychological and behavioral development. Consequently, intensive parenting may be associated with college students through both positive mechanisms, such as academic monitoring, learning resource support, and emotional encouragement, and negative mechanisms, including excessive control, restriction of autonomy, and the transmission of achievement pressure (Linkiewich et al., 2021). Through these processes, intensive parenting directly shapes students' academic self-management abilities (e.g., time management, task execution, and emotional regulation) and goal orientation, thereby demonstrating the direct influence of environmental factors on individuals' internal psychological mechanisms (Mulisa, 2019).

Academic self-management and goal orientation function as mediating mechanisms linking environmental influences to developmental outcomes and play critical transformative roles within the proposed model (Martínez-López et al., 2024). From the perspective of Ecological Systems Theory, environmental influences, such as family expectations and teachers' academic requirements, cannot be directly translated into developmental outcomes (Yang, 2023). Instead, these influences first shape individuals' goal-related cognitions, including whether they prioritize developing personal competence, avoid challenges because of fear of failure, or perceive academic success as a means of fulfilling parental expectations. These goal-related cognitions are subsequently is associated with students' academic self-management strategies, including whether they proactively develop individualized learning plans, persist in learning without external supervision, and adapt learning strategies flexibly to changing academic demands. Ultimately, these internal mechanisms are reflected in differences in academic performance and psychological adjustment (Tepordei et al., 2025). Accordingly, the influence of perceived intensive parenting on college students' academic achievement is primarily transmitted through these internal mechanisms of goal orientation and academic self-management rather than through a simple direct effect (Yu and Xu, 2022).

### 2.2 The relationship between intensive parenting and academic achievement

Zhou Z. et al. (2025) mentioned that intensive parenting by parents can significantly enhance children's cognitive and non-cognitive abilities, strengthen their competitive edge in education, and thereby drive academic achievement improvement. Rodiyah et al. (2025) suggest that the high level of educational attention, clear expectations, targeted guidance, and appropriate supervision exhibited by parents during intensive parenting are beneficial for children in forming good learning habits and methods, thereby ensuring academic success. Tang et al. (2024) further discovered that parents who adopt intensive family education show high levels of involvement, systematization, and organization in areas such as academic support, parent-child companionship, family language use, and the distribution of parenting responsibilities, which significantly promotes children's cognitive intelligence, academic scores, interests, and talents in various aspects. Abdillah and Merdiaty (2025) suggest that if parents excessively monitor and intervene in their children's learning process, it may restrict their space for independent thinking and fail to meet their need for autonomous exploration, ultimately having a negative impact on their academic achievement. However, this situation can be avoided by appropriately balancing the intensity and boundaries of parenting.

Non-Chinese studies have further clarified the effectiveness, pathways, and stability of the relationship between the two, providing cross-cultural evidence for the reasonableness of the hypothesis: In terms of mechanisms, Qian (2024) and Hayek et al. (2022) have both verified that intensive parenting can promote academic achievement through dual pathways. In the direct pathway, clear rule-setting and emotional support can directly improve academic achievement; in the indirect pathway, it can meet children's needs for competence and autonomy, enhance self-esteem, self-efficacy, and academic intentions, thereby increasing the degree and persistence of academic engagement, ultimately leading to excellent academic achievement. Fute et al. (2024) further added that learning engagement (across emotional, cognitive, and behavioral dimensions) is an important mediating variable, and intensive parenting can significantly improve academic scores by stimulating learning engagement. In summary, based on existing research conclusions, this study proposes the following hypothesis:

1. H1: Chinese college students perceive that intensive parenting by their parents is positively associated with on their academic achievement.

### 2.3 The mediating role of academic self-management

Intensive parenting provides a wealth of learning resources, high involvement in academics, and clear expectations for academic achievement. These are the core characteristics that build an external support framework for children's academic progress. However, for this framework to truly take effect, it largely depends on the student's ability to manage their own academic work. This ability becomes an important intermediary linking intensive parenting and academic achievement (Hayek et al., 2022).

From the perspective of resource conversion, the tutoring materials, extracurricular tutoring, and academic connections provided by parents under intensive parenting need to be accurately utilized through academic self-management (Komlavathi and Yasin, 2022). Students with strong self-management skills can leverage the planning of learning goals and processes to deeply align external resources with their academic weaknesses and course progress. For example, they can prioritize using tutoring materials to tackle weaker subjects and reasonably schedule time for extracurricular tutoring and self-study, maximizing the efficiency of resource use (Komlavathi and Yasin, 2022). This way, external support can be transformed into intrinsic academic advantages. Intensive parenting often comes with high academic expectations, which can easily lead to implicit psychological pressure. However, the emotional regulation aspect of academic self-management can effectively buffer this pressure: students can maintain a positive learning mindset by objectively assessing whether the expectations are reasonable, breaking down goals into manageable stages, and self-motivating. This helps avoid anxiety from interfering with their learning state and maintain academic resilience in a high-pressure environment (Soge et al., 2025).

Empirical research has further verified the stability and general applicability of this mediating mechanism. Affuso et al. (2023) conducted a longitudinal study that found when parents express their emphasis on education through academic supervision, it not only meets adolescents' basic psychological needs for autonomy and competence but also enhances self-determined motivation and academic self-efficacy. These core elements of self-management can, through optimized learning strategies and increased task engagement, indirectly promote academic achievement. Moreover, this pathway remains stable even after controlling for confounding variables such as intelligence and parents' education levels. Xiao et al. (2025) also confirmed that parents, through academic guidance, behavioral supervision, and positive feedback, can help adolescents establish a regular study rhythm, enhance self-assessment and task execution abilities, and form efficient self-management strategies that include time allocation, sleep optimization, and self-reflection. Moreover, the self-management habits cultivated by this parenting style are persistent and have a significant positive indirect effect on academic achievement across different semesters. Hayek et al. (2022) conducted a study on intensive parenting (authoritative type) and revealed a serial mediation pathway: intensive parenting first positively enhances academic self-management abilities, and high self-management further strengthens the intention to achieve good grades, with both factors synergistically promoting academic achievement. Based on this, this study proposes the hypothesis:

1. H2: The self-management of academic studies among Chinese college students mediates the relationship between perceived intensive parenting and academic achievement.

### 2.4 The mediating role of goal orientation

Goal orientation plays a crucial role between intensive parenting by parents and children's academic achievement, becoming the core transmission mechanism connecting the two aspects. Existing research has confirmed that the impact of parenting styles on students' academic achievement is not direct but mainly achieved by shaping students' goal orientation (Ye et al., 2024). In an intensive parenting environment, children, in responding to their parents' academic expectations and pursuing the learning goals set by the family, gradually develop a stable mastery goal orientation or performance goal orientation. These two different orientations, through influencing learning attitudes, method choices, and intrinsic motivation, ultimately affect academic achievement (Xu et al., 2024).

The study by Asanjarani et al. (2022) further verifies that the positive aspects of intensive parenting, such as warm support and emotional understanding, can effectively stimulate children's interest in learning and intrinsic motivation, allowing them to see learning as a process of self-improvement rather than merely completing tasks. This, in turn, establishes a lasting mastery-oriented goal. Additionally, by setting clear learning goals and providing continuous encouragement, parents can guide children to internalize external expectations into their own pursuits, prompting them to actively challenge themselves in their studies. Once goal orientation is established, children will more consciously optimize their learning strategies, maintain learning resilience, and enhance their learning abilities through continuous self-improvement. This ultimately leads to a significant increase in academic achievement, fully demonstrating the pathway through which parenting styles are associated with academic outcomes via goal orientation (Hayek et al., 2022).

Research related to Chinese populations also provides strong support: Xu et al. (2024) focused on Chinese adolescents and found that parental psychological control, as a negative manifestation of intensive parenting, exacerbates maladaptive academic behaviors in children by reinforcing performance-approach and performance-avoidance goal orientations. In contrast, parental autonomy support reduces such maladaptive behaviors by fostering mastery goal orientation, and this influence pathway is not affected by cultural background, demonstrating cross-cultural consistency. Chen and Mok (2023) also found that intensive parenting by parents (including family tutoring, school communication, academic socialization, etc.) is significantly positively correlated with mastery goal orientation and performance approach goal orientation. Moreover, these two types of goal orientations partially mediate the positive relationship between parenting and academic buoyancy and adaptability, helping students better cope with learning challenges. Caparello et al. (2025) further revealed in their study on college students that a mother's mastery goal orientation can enhance her child's mastery goal orientation, thereby increasing their academic satisfaction and indirectly promoting academic achievement. This study proposes the following hypothesis:

1. H3: The goal orientation of Chinese college students mediates the relationship between perceived intensive parenting and academic achievement.

## 3 Research methods

### 3.1 Research framework

**Figure 1:**
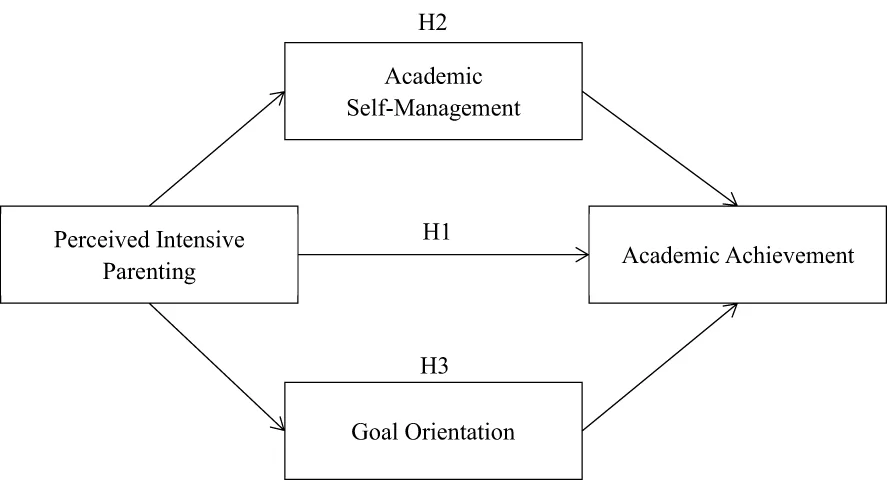
Research framework diagram.

The research framework diagram clearly presents the relationships between the variables and the research direction. As shown in Figure 1.

### 3.2 Research methods and research subjects

This study distributed online surveys through Wenjuanxing, targeting Chinese college students. Using random sampling, six different types of undergraduate institutions from the eastern, central, and western regions of China were selected for the survey. These included comprehensive universities, normal universities, and science and engineering universities, ensuring the uniformity of university type sampling.

This study collected a total of 569 questionnaires, investigating the background information of Chinese university students, including gender, grade, whether they are only children, and the average number of times they contact their parents per week. The results show: in terms of gender, there were 264 male students (46.397%) and 305 female students (53.603%); in terms of grade, there were 63 freshmen (11.072%), 178 sophomores (31.283%), 165 juniors (28.998%), and 163 seniors (28.647%); In terms of the only child status, there are 279 only children (49.033%) and 290 non-only children (50.967%); regarding the average number of times they contact their parents each week, 27 people (4.745%) do not contact their parents, 301 people (52.900%) contact them 1–2 times, and 241 people (42.355%) contact them 3 times or more.

### 3.3 Research tools

#### 3.3.1 College students' perception of intensive parenting scale

Based on a comprehensive review of relevant measurement tools both domestically and internationally, this study developed the “College Students' Perception of Intensive Parenting Scale.” The scale is primarily based on the IPAQ proposed by Liss et al. (2012), as well as the measurement frameworks of Gauthier et al. (2021) and Yi and Cheng (2021), and appropriate cultural adjustments and content revisions were made in conjunction with the growth context of Chinese college students. To ensure the content validity of the scale, this study invited five experts in the fields of education and psychology to conduct multiple rounds of evaluation and revision of the items, and calculated the Item-level Content Validity Index (I-CVI), with results all being 1.00, indicating that the items have good content representativeness. The final formal scale consists of 4 dimensions and 19 items, namely child-centered, resource-intensive, emotional investment, and expert guidance. A sample item is: “My parents communicate with me at least once a week about my school affairs, learning progress, living conditions, and social interactions.” The scale uses a 5-point Likert scoring system (1 indicates very disagree, 5 indicates very agree).

#### 3.3.2 Academic achievement scale

This study uses the academic achievement scale developed by Luo et al. (2023) to measure the academic achievement levels of college students. The scale contains a total of 12 items, primarily evaluated from four aspects: academic engagement, learning performance, interpersonal promotion, and objective achievement, with each dimension including 3 items. One of the items in this scale is: “I am enthusiastic about the courses in my major and actively devote a significant amount of time to studying.” All items are scored using a 5-point Likert scale, with a range from “1 = strongly disagree” to “5 = strongly agree.” The higher the score, the higher the student's academic achievement level.

#### 3.3.3 Academic self-management scale

This study uses the Academic Self-Management Scale developed by Tangney et al. (2004) as the measurement tool. The scale consists of 13 items and has a unidimensional structure, used to assess students' self-management abilities during the learning process. A representative item of the scale is: “I can proactively complete learning tasks on time and honor my study commitments.” All items are scored using a 5-point Likert scale, with a range from “1 = Strongly Disagree” to “5 = Strongly Agree.” The higher the score, the higher the student's academic self-management level.

#### 3.3.4 Goal orientation scale

This study uses the Claremont Purpose Scale (CPS) developed by Bronk et al. (2018) to measure goal orientation. The scale includes three dimensions: personal meaning, investment and effort, and transcendence of self, with 4 items set for each dimension. A sample item is: “I am highly engaged when carrying out the plans I have set for myself.” All items are scored using a 5-point Likert scale, where 1 indicates “strongly disagree” and 5 indicates “strongly agree.” Higher scores indicate a higher level of goal orientation for the individual.

#### 3.3.5 Control variables

To eliminate other exogenous factors that may be associated with the relationship between perceived intensive parenting and academic achievement among university students, this study controls for four variables. First, the frequency of weekly contact between university students and their parents is controlled, as prior research has indicated that such contact may affect students' academic achievement (Kumah et al., 2024). Second, gender is controlled, given that previous studies have shown that female students tend to achieve higher academic performance than male students (Bowman et al., 2022). Additionally, only-child status is included as a control variable, as evidence suggests that only children may demonstrate superior academic performance (Jia et al., 2022). Finally, students' academic year is incorporated as a control variable, as differences in academic year may be associated with academic achievement (Khan et al., 2024). In this context, this study includes the frequency of weekly contact with parents, gender, academic year, and only-child status as control variables.

### 3.4 Research ethics

This study was conducted in accordance with the requirements of Articles 20 and 21 of Section 3.2.2 of the 2015 version of the National Policy and Guidelines for Human Research. The research protocol was reviewed and ethically approved by Dhurakij Pundit University in Thailand (DPU_BSH 1807/2567) on July 18, 2025. During the research process, the researchers strictly protected participants' personal information and privacy, ensuring that all participants voluntarily participated in the study. Participants were informed that they had the right to withdraw from the study at any time without any negative consequences if they felt uncomfortable.

Prior to data collection, approval for conducting the survey was obtained from the relevant authorities of the participating universities in September 2025. The data collection was conducted among faculty members from six universities in China, including Zhejiang University, Shanghai Jiao Tong University, Central China Normal University, Central South University, Lanzhou University, and Yunnan University.

In addition, the research team placed the highest priority on respecting and protecting participants' personal information and privacy. An informed consent form was carefully developed, clearly explaining the purpose, procedures, and voluntary nature of the study. Participants independently decided whether to sign the informed consent form to indicate their willingness to participate. All information collected in this study was handled with strict confidentiality and ensured accuracy, authenticity, and integrity.

## 4 Research results

### 4.1 Common method bias

This study employed Harman's single-factor test to examine the potential issue of common method bias. All measurement items were entered into an unrotated principal component analysis. The results showed that 18 factors with eigenvalues greater than 1 were extracted. The first factor accounted for 37.020% of the total variance, which is well below the recommended threshold of 50% (Podsakoff et al., 2003). In this context, common method bias was not considered a serious concern in this study, providing preliminary support for the reliability and validity of the research findings. The results of the common method bias test are presented in Table 1.

**Table 1:** Summary of common method bias test results.

Factor	Variance explained (%)	Cumulative variance explained (%)
1	37.020	37.020
2	10.248	47.268
3	5.000	52.268
4	3.999	56.267
5	3.006	59.273
6	2.129	61.402
7	1.876	63.279
8	1.836	65.115
9	1.608	66.723
10	1.487	68.210
11	1.372	69.581
12	1.347	70.929
13	1.293	72.222
14	1.261	73.483
15	1.213	74.696
16	1.123	75.819
17	1.087	76.905
18	1.043	77.948

### 4.2 Reliability analysis

The Cronbach's Alpha value of the Perceived Intensive Parenting Scale is 0.945, the Cronbach's Alpha value of the academic achievement scale is 0.897, the Cronbach's Alpha value of the academic self-management scale is 0.954, the Cronbach's Alpha value for the goal orientation scale is 0.914, indicating that the overall questionnaire has high internal consistency and measurement stability (see Table 2).

**Table 2:** Reliability analysis table.

Dimensions	Items	Cronbach's alpha
Perceived intensive parenting	19	0.945
Academic achievement	12	0.897
Academic self-management	13	0.954
Goal orientation	12	0.914

### 4.3 Validity analysis

To test the convergent validity of each dimension of the scale, this study used confirmatory factor analysis to evaluate factor loadings, composite reliability, and average variance extracted. In the child-centered dimension, the standardized factor loadings of each item range from 0.709 to 0.853, with a CR value of 0.911 and an AVE value of 0.632; in the resource-intensive dimension, the factor loadings range from 0.638 to 0.850, with a CR value of 0.898 and an AVE value of 0.598. The factor load range for the emotional investment construct is 0.665 to 0.798, with a CR value of 0.849 and an AVE value of 0.585. The factor load range for the expert guidance construct is 0.659 to 0.891, with a CR value of 0.838 and an AVE value of 0.637. The factor loadings of the items in the academic engagement dimension range from 0.752 to 0.791, with a CR value of 0.815 and an AVE value of 0.595; the factor loadings in the academic achievement dimension range from 0.741 to 0.797, with a CR value of 0.811 and an AVE value of 0.589; the factor loadings in the interpersonal promotion dimension range from 0.706 to 0.766, with a CR value of 0.783 and an AVE value of 0.546; the factor loadings in the objective achievement dimension range from 0.685 to 0.755, with a CR value of 0.769 and an AVE value of 0.526. The standardized factor loadings of the items for academic self-management range from 0.705 to 0.857, with a CR value of 0.954 and an AVE value of 0.617. The factor loadings of the items in the personal significance dimension range from 0.718 to 0.831, with a CR value of 0.870 and an AVE value of 0.627; the factor loadings in the engagement and effort dimension range from 0.709 to 0.842, with a CR value of 0.855 and an AVE value of 0.596; the factor loadings in the self-transcendence dimension range from 0.649 to 0.859, with a CR value of 0.874 and an AVE value of 0.637. The overall convergent validity meets the requirements. In summary, the factor loadings of the items in each dimension are all greater than 0.500, the CR values are not less than 0.600, and the AVE values are greater than 0.500. This shows that the dimensions of the four scales have good convergent validity, and the scale structure is reasonable.

### 4.4 Structural equation model analysis

Based on the confirmation of good structural validity through confirmatory factor analysis of the measurement model, this study constructed a parallel multiple mediation structural equation model, with perceived intensive parenting as the independent variable, academic achievement as the dependent variable, and academic self-management and goal orientation as parallel mediating variables. The model was used to examine both the direct effect of the independent variable on the dependent variable and the two independent mediating effects.

#### 4.4.1 Model fit analysis

The model fit results showed that the model demonstrated an acceptable fit to the data, with χ^2^/df = 4.061, CFI = 0.920, TLI = 0.911, RMSEA = 0.073, and SRMR = 0.071. Overall, the χ^2^/df value was below 5, both the CFI and TLI values exceeded 0.900, and the RMSEA and SRMR values were below 0.080. All fit indices met the recommended criteria, indicating that the proposed model fit the observed data well and was suitable for subsequent path analysis and hypothesis testing.

#### 4.4.2 Path analysis

The results of the structural equation model path analysis are presented in Table 3. All hypothesized paths were statistically significant (*p* < 0.001). Specifically, perceived intensive parenting significantly and positively predicted academic self-management (standardized path coefficient β = 0.565, C.R. = 12.041) and goal orientation (standardized path coefficient β = 0.658, C.R. = 13.115). Academic self-management significantly and positively predicted academic achievement (standardized path coefficient β = 0.219, C.R. = 5.359), while goal orientation also exerted a significant positive effect on academic achievement (standardized path coefficient β = 0.555, C.R. = 9.526). In addition, perceived intensive parenting had a significant direct positive effect on academic achievement (standardized path coefficient β = 0.247, C.R. = 4.308). Taken together, the results show that perceived intensive parenting can directly and positively be associated with academic achievement. Based on these observations, H1 was supported.

**Table 3:** Results of path analysis.

Path	Estimate	Std. estimate	**S.E**.	**C.R**.	***p***
Perceived intensive parenting → academic self-management	0.474	0.565	0.039	12.041	< 0.001
Perceived intensive parenting → goal orientation	0.546	0.658	0.042	13.115	< 0.001
Academic self-management → academic achievement	0.209	0.219	0.039	5.359	< 0.001
Goal orientation → academic achievement	0.537	0.555	0.056	9.526	< 0.001
Perceived intensive parenting → academic achievement	0.198	0.247	0.046	4.308	< 0.001

#### 4.4.3 Mediation analysis

The mediating effects of the parallel multiple mediation model were examined using the Bootstrap method with 5,000 resamples. The results are presented in Table 4.

**Table 4:** Bootstrap analysis of mediation effects.

Path	Effect	SE	LLCI	ULCI
Total effect	0.735	0.034	0.662	0.798
Direct effect	0.247	0.057	0.138	0.359
Indirect effect 1	0.124	0.037	0.055	0.203
Indirect effect 2	0.365	0.061	0.253	0.493

Bootstrap resampling was performed 5,000 times.

LLCI, lower limit of the 95% confidence interval; ULCI, upper limit of the 95% confidence interval.

Indirect effect 1 = perceived intensive parenting → academic self-management → academic achievement; indirect effect 2 = perceived intensive parenting → goal orientation → academic achievement.

The total effect of perceived intensive parenting on academic achievement was 0.735 (SE = 0.034), with a 95% confidence interval of [0.662, 0.798]. With the confidence interval not including zero, the total effect was statistically significant. The direct effect was 0.247 (SE = 0.057), with a 95% confidence interval of [0.138, 0.359], which also excluded zero. This finding indicates that perceived intensive parenting exerted a significant direct positive effect on academic achievement, supporting H1.

The indirect effect of the pathway perceived intensive parenting → academic self-management → academic achievement was 0.124 (SE = 0.037), with a 95% confidence interval of [0.055, 0.203]. Because the confidence interval did not include zero, the independent mediating effect of academic self-management was significant, supporting H2.

The indirect effect of the pathway perceived intensive parenting → goal orientation → academic achievement was 0.365 (SE = 0.061), with a 95% confidence interval of [0.253, 0.493]. As the confidence interval excluded zero, the independent mediating effect of goal orientation was significant, supporting H3.

Combined with the path coefficient results reported above, the confidence intervals of both indirect pathways did not include zero, and the direct effect remained significant. These findings further confirm that academic self-management and goal orientation play parallel partial mediating roles in the relationship between perceived intensive parenting and academic achievement.

## 5 Discussion

### 5.1 The significant positive effect of perceived intensive parenting on Chinese college students' academic achievement

The findings indicate that perceived intensive parenting significantly and positively predicts Chinese college students' academic achievement, thereby supporting H1. This result is consistent with the findings of Yang (2024), who reported that intensive parenting and parental emotional warmth facilitate academic achievement. As a typical interaction pattern within the family microsystem, intensive parenting provides multidimensional support for students' academic development through high levels of parental investment and involvement, thereby directly promoting academic achievement (Du and Li, 2024). Within the traditional Chinese cultural context that emphasizes high parental expectations for children's success, together with the increasingly competitive educational environment, the positive effects of intensive parenting become particularly evident. Through resource investment and emotional encouragement, parents create a well-structured and highly supportive learning environment that fosters college students' academic development (Sun et al., 2023).

### 5.2 The mediating role of academic self-management among Chinese college students

The results demonstrate that academic self-management shows mediating associations in perceived intensive parenting and academic achievement among Chinese college students, supporting H2. This finding extends the understanding of the sequential pathway linking family parenting, individual psychological characteristics, and academic achievement (Li et al., 2023). Specifically, the resource support, academic supervision, and emotional encouragement provided through intensive parenting contribute to the development of students' academic self-management by transforming external parental supervision into autonomous time-management awareness and converting family resource support into effective learning behaviors (Wang et al., 2023). These findings provide important implications for strengthening collaboration between family and higher education. Families should actively cultivate their children's self-management abilities, while universities should systematically implement academic self-management training. Only through the joint efforts of both parties can family parenting resources be effectively translated into improved academic achievement (Song et al., 2024).

### 5.3 The mediating role of goal orientation among Chinese college students

The findings further reveal that goal orientation mediates the relationship between perceived intensive parenting and academic achievement among Chinese college students, thereby supporting H3. This result further enriches the understanding of the mediating mechanism linking family parenting, individual psychological characteristics, and academic achievement (Lin, 2024). As a core motivational factor, goal orientation encourages college students to actively engage in academic activities. In other words, students are more likely to participate proactively in research and practical learning to enhance their competencies, while maintaining sustained and efficient learning behaviors to achieve their academic goals, ultimately contributing to higher academic achievement (Chen and Mok, 2023). These findings suggest that the influence of intensive parenting extends beyond fostering behavioral self-management and more fundamentally operates through enhancing students' goal orientation at the motivational level, thereby promoting their academic development (Sun, 2025).

## 6 Research conclusions

Research results indicate that Chinese college students' perception of intensive parenting by their parents is positively associated with academic achievement, with academic self-management and goal orientation both playing mediating roles between the perception of intensive parenting and academic achievement. It can be seen that parenting styles are not only associated with college students' academic achievement but also indirectly promote academic development through individual psychological mechanisms. Based on the results, the hypotheses H1, H2, and H3 proposed in this study are all supported.

## 7 Research contributions

On a theoretical level, this study expands the scope of research on intensive parenting. Previous related studies have mostly focused on the group of primary and secondary school students, while this study extends the research subjects to the university student stage, thereby providing new evidence for understanding the impact of family upbringing on the long-term academic development of individuals (Brooks et al., 2025; Cui et al., 2022). At the same time, this study examined the positive effects of intensive parenting on academic development within the context of Chinese culture, further enriching cross-cultural research on the relationship between parenting styles and academic achievement (Du and Li, 2024). In addition, this study incorporates academic self-management and goal orientation as mediating variables to examine the psychological mechanisms through which perceived intensive parenting is associated with academic achievement. Rather than directly testing ecological systems theory, the findings are broadly consistent with its proposition that environmental is associated with operate through individuals' internal psychological processes (Chen and Mok, 2023; Gandarillas et al., 2024; Zaatari and Maalouf, 2022). Accordingly, this study extends previous research by integrating parenting practices and individual psychological factors into a single analytical model for understanding college students' academic achievement.

On a practical level, this study provides valuable insights for family education and higher education. The research results indicate that parents providing continuous emotional support, academic communication, and resource investment during the university stage are positively associated with students' academic development, but excessive control may have negative effects (Du and Li, 2024; Yan et al., 2025). Given these findings, family education should place greater emphasis on balancing support and autonomy, and gradually transition from experience-based to scientific and precise parenting methods (Jensen et al., 2024). At the same time, universities should emphasize the critical role of academic self-management and goal orientation in academic development, enhancing students' autonomous learning abilities through academic planning courses, self-management training, and career guidance (Simón-Grábalos et al., 2025; Xu et al., 2024). In addition, the research also emphasizes the synergistic effect between families and universities, promoting the academic development of college students through enhanced communication and information sharing between home and school, thereby providing practical references for improving educational quality and equity (Lu and Sarmiento, 2024; Li, 2025).

## 8 Research recommendations

First, parents should be guided to adopt a more balanced intensive parenting approach during their children's university years by adjusting different dimensions of intensive parenting according to students' developmental needs. In terms of the child-centered dimension, parents should pay attention to students' academic and emotional needs while respecting their autonomy and avoiding excessive intervention in academic and life decisions, thereby fostering independent learning and academic self-management. Regarding the resource-intensive dimension, parents should provide appropriate educational resources, such as learning materials, academic information, and opportunities for skill development, rather than relying solely on increased financial investment. For the emotional investment dimension, parents should offer emotional encouragement and understanding when students encounter academic stress, helping them maintain confidence and a stronger sense of purpose in learning rather than focusing exclusively on academic performance. Finally, within the expert guidance dimension, parents should provide advice on academic planning and career development while encouraging students to make independent decisions, thereby supporting the development of long-term learning goals and personal responsibility.

Secondly, the academic self-management skills of college students should be further enhanced. College students need to proactively establish clear study plans, reasonably allocate time according to personal goals and course requirements, and break down long-term goals into executable phase tasks. At the same time, continuous self-monitoring and reflection should be conducted during the learning process, and learning strategies should be adjusted promptly based on feedback. Colleges can also provide necessary support to students through academic counseling and learning strategy guidance, helping them transform external resources into sustained learning motivation.

Finally, the development of goal-oriented approaches for college students should be strengthened. College students should combine their own interests, professional characteristics, and future development plans to establish a clear and hierarchical learning goal system, and break down long-term goals into short-term ones. During the implementation of goals, one can enhance learning motivation through periodic summaries and self-motivation, and maintain continuous effort when encountering difficulties. At the same time, students should actively communicate with parents, teachers, and peers, continuously adjusting their learning direction through diverse feedback, thereby forming a more stable and sustainable goal orientation.

## 9 Limitations of the study and suggestions for future research

This study employs a cross-sectional questionnaire design, which can only reflect the static relationship of variables at a single point in time, making it difficult to reveal the long-term effects and causal mechanisms of intensive parenting on college students' academic development and psychological adaptation. Future research could introduce longitudinal tracking designs to continuously observe relevant variables, thereby better examining their dynamic changes and causal relationships. Secondly, the research methods mainly rely on quantitative self-reported questionnaires, and all variables were collected from the same respondents at a single point in time. Although the common method bias test indicated that common method bias was not a serious concern, the possibility of common method variance cannot be completely ruled out. In addition, a single method is insufficient to fully capture the complex interactions between variables, especially in dimensions such as implicit emotional transmission and individual subjective experiences involved in the parenting process, which still lack refined measurement. Self-reported data may also be influenced by social desirability and subjective perceptions. It is recommended that future research combine qualitative and quantitative studies to delve deeper into the intrinsic mechanisms of intensive parenting within the context of local culture, collect data from multiple sources and at multiple time points, and focus on the changes in parent-child interaction methods in the digital age and their associations with college students' development. In addition, future research should strengthen the practical application and evaluation of research findings by building a collaborative support system between home and school, and by using experimental or quasi-experimental designs to test the effectiveness of interventions, thereby enhancing the practical value of the research. Although this study was grounded in Ecological Systems Theory, the empirical model primarily focused on the microsystem by examining perceived intensive parenting and individual psychological mechanisms. The meso-, exo-, and macro-system factors proposed by the theory were not incorporated into the analytical model. Therefore, the present study reflects only part of the ecological framework rather than testing the theory comprehensively. Future research could integrate multiple ecological levels, such as school, peer, community, and sociocultural contexts, to provide a more comprehensive examination of college students' academic development.

## Data Availability

The raw data supporting the conclusions of this article will be made available by the authors, without undue reservation.
